# Famous Poisoners in Fiction

**Published:** 1893-09-02

**Authors:** 


### Famous Poisoners in Fiction.

### IV.—A ROYAL POISONER.

In the sixteenth century the art of poisoning reached its most perfect point. Since then it has gradually declined until now we are obliged, as in the art of painting, to turn to the "Old Masters" of this cruel handicraft for its best examples, its most successful essays. To-day this art is no longer made a study, although even in this more enlightened century there are still to be found in the dark places of the earth able followers of Réné, Hatton, Catherine de Medici, &c.

People love to talk about the "Good old days;" bad old days they might better be called, for then wickedness and sin was rampant, and death reigned on every side. Then brave men wore mail shirts beneath their doublets for fear of the dagger's point, and the dread of poison haunted even the boldest, the most intrepid; for against this subtlest of foes neither valour nor prudence were of aught avail. In those days, when men held other men's lives lightly; when death seemed but a slight revenge; when a poor man swung for stealing a rabbit; when fatal duels were a daily occurrence, the poisoner poisoned for gain, for fame, for advancement, and the chemist studied the science of medicine, not to save the life of his patients, but to bring about secretly the destruction of his own or of his employer's victims.

Historical novels give to their characters motives and reasons which may or may not have swayed the hearts of the long dead, yet once living, personages portrayed in the story; truth and fiction are ever mixed amazingly therein. But, at least, they are founded on fact; the names, the outlines are true, even if the imagination has been used to fill in these word-pictures of other days and other manners. "Margaret of Navarre"* deals with Court life in the days of Charles IX. of France. That mystery enwraps the death of this young King is a known fact, and there is little doubt he was believed at the time to have been unintentionally poisoned. How much truth there is in the theory of the book, the leaves of which had been poisoned, and which, intended for another, was handled unwittingly by the ill-starred monarch, can never be known until the secrets of the ages be revealed. But that he met his death in a strange manner is certain, and his illness had every symptom of poisoning by arsenic; but, on the other hand, it should be remembered that in those days when poisoning was a trade, and folks eyed sharply and suspiciously their fellow men, no Royal death—and especially the death of a youthful Sovereign—could occur without mysterious and malicious whispering. But that all the later Medicis, and Catherine above all, made use of poison to rid themselves of national or individual enemies, is practically proved. A restless, ambitious, intriguing woman, with a head filled with dark designs and bitter malignity—a cruel, implacable foe, a dangerous friend — Catherine de Medici was worthy of her race and name. That she had an able tool in Réné is also true. In the age in which these two subtle destroyers lived the Court poisoner was as necessary as the Court doctor is to-day. There is a sort of devilry in this man Réné, the poisoner by profession, which makes the reader shudder whilst he reads; no hatred stirs his veins, no revenge lights his eye, goading him on like an evil genius. Slowly, in the most matter of fact, business-like manner, he deals out death to a known or unknown victim. But there is one thing haunts him—*i.e.*, fear of punishment if discovered. Eagerly he questions both the King and Henry of Navarre, if confession will bring pardon; on this condition, and this only, does he confess!

Poison in the middle ages, when poisoning was a very common matter, was introduced into nearly everything. It is rumoured that Jeanne of Navarre came by her death by means of a poisoned pair of gloves, and Dumas makes Réné tell how that the very cosmetics the Court ladies used, the very oil burnt in the bed-chamber lamp contained ofttimes the elements of death—aye, even the very books read for amusement were made the instrument for removing the unhappy reader from this nether world—a world as full of danger, as full of care, if not quite so weary, quite so disappointing, quite so cynical as that of to-day.

In this and the next century the art of poisoning was perfected, but so terrible was the result, so awful became the tension of a life of con-tinual suspicion and dread that, as it were, by mutual if silent consent, a compact seemed made by man and man, and a reaction set in which has lasted till the present day, and thus the office of Court poisoner has fallen into disuse and abeyance, and though men still poison for private advancement or revenge, politically the use of poison as a defensive or offensive weapon has long been abandoned in at least all European countries. In the East, *on dit*, arsenic is still sometimes mingled in the coffee so courteously offered to the offending Minister of State, who dare not refuse to drink, just as the Italian nobles dared not refuse to dine with Pope Alexander VI., even although they knew death lurked in the tempting viands.

Arsenic is the poison which Alexander Dumas (*père*) suspects that the book was impregnated with; for arsenic then, as now, was apparently one of the favourite poisons of those who wished (or whose enemies wished) that they were dead. The effect of the fire on the arsenic is graphically and truly described. The greenish blue flame, as it points upwards, testifies to the guilty deed; the intended victim has escaped, but the dread tyrant, Death, will have its prey; this time it is a crowned King he claims, the wretched son of the royal poisoner! Verily, crimes as well as curses come back to roost. One word in conclusion about the Queen mother, whose character this historical romance delineates in true if awful blackness. During the reigns of her husband and three sons Catherine of Medici ruled France, her sceptre poison, her rule death to all who would not become her tools and bow beneath her sway. Here is an historical piece of evidence to prove that poison is woman's "frenzied woman's" keened weapon and the "gay deceiver's" keenest arrow. The impulse of the brute force in man is to strike, the cunning wit of woman bids her destroy secretly, surely, silently. If woman is a more devoted friend, a more self-sacrificing lover than man, she is also a more dangerous, more intensely cruel foe. It seems to me that there is some truth in the old theory that man represents the earth and woman either heaven or hell, for if stronger and more faithful in love she is also more daring, more determined, more cruel in hate.

* "Margaret of Navarre," by Alexander Dumas (*père*), author of "The Count of Monte Christo," &c.

